# Gestational age, mode of birth and breastmilk feeding all influence acute early childhood gastroenteritis: a record-linkage cohort study

**DOI:** 10.1186/s12887-016-0591-0

**Published:** 2016-04-27

**Authors:** Jason P. Bentley, Judy M. Simpson, Jenny R. Bowen, Jonathan M. Morris, Christine L. Roberts, Natasha Nassar

**Affiliations:** Clinical and Population Perinatal Health Research, Kolling Institute, University of Sydney, Sydney, NSW Australia; Sydney School of Public Health, University of Sydney, Sydney, NSW Australia; Department of Neonatology, Royal North Shore Hospital, Sydney, NSW Australia; University Department of Obstetrics, Building 52, Royal North Shore Hospital, St Leonards, NSW 2065 Australia

**Keywords:** Acute gastroenteritis, Early term birth, Caesarean section, Child, Healthy start to life, Breastfeeding

## Abstract

**Background:**

Acute gastroenteritis (AGE) is a leading cause of infectious morbidity in childhood. Clinical studies have implicated caesarean section, early birth and formula feeding in modifying normal gut microbiota development and immune system homeostasis in early life. Rates of early birth and cesarean delivery are also increasing worldwide. This study aimed to investigate the independent and combined associations of the mode and timing of birth and breastmilk feeding with AGE hospitalisations in early childhood.

**Methods:**

Population-based record-linkage study of 893,360 singleton livebirths of at least 33 weeks gestation without major congenital conditions born in hospital, New South Wales, Australia, 2001–2011. Using age at first AGE hospital admission, Cox-regression was used to estimate the associations for gestational age, vaginal birth or caesarean delivery by labour onset and formula-only feeding while adjusting for confounders.

**Results:**

There were 41,274 (4.6 %) children admitted to hospital at least once for AGE and the median age at first admission was 1.4 years. Risk of AGE admission increased with decreasing gestational age (37–38 weeks: 15 % increased risk, 33–36 weeks: 25 %), caesarean section (20 %), planned birth (17 %) and formula-only feeding (18 %). The rate of AGE admission was highest for children who were born preterm by modes of birth other than vaginal birth following the spontaneous onset of labour and who received formula-only at discharge from birth care (62–78 %).

**Conclusions:**

Vaginal birth following spontaneous onset of labour at 39+ weeks gestation with any breastfeeding minimised the risk of gastroenteritis hospitalisation in early childhood. Given increasing trends in early planned birth and caesarean section worldwide, these results provide important information about the impact obstetric interventions may have on the development of the infant gut microbiota and immunity.

**Electronic supplementary material:**

The online version of this article (doi:10.1186/s12887-016-0591-0) contains supplementary material, which is available to authorized users.

## Background

Acute gastroenteritis is characterised by viral or bacterial infection causing diarrhea and vomiting and is a leading cause of infectious morbidity in infants and children worldwide even in developed countries including Australia, where the incidence is highest in the first two years of life [1, 2]. Many factors in early childhood are associated with an increased susceptibility to gastroenteritis, such as poor social conditions, diet and antibiotic use [3, 4]. Additionally, gut microbial composition and immunological immaturity in the newborn may also play an important role [5–8].

Bacterial exposures from the birth canal and perianal region during vaginal birth are important precursors for the colonisation of the gut in the first few days of life. To prepare for this, cells of the adaptive immune system are recruited to the fetal intestinal tissue with a transition to adult T-cells occurring in the third trimester [5]. Once born, a multitude of pathways activate to prepare the immune system and intestinal epithelial cells to manage the high density of bacteria in the gut, establishing a homeostasis between the immune system and gut microbiota [6]. The later the gestational age at birth, the better prepared the newborns immune system is for establishing homeostasis. Bacterial colonisation and the immune response in the gut are further supported by exposure to the nutritional, growth and immunological factors contained in breastmilk [7]. Clinical studies have shown gut colonisation is typically imbalanced towards bacterial species such as *E. Coli.* in infants delivered by caesarean section or fed formula rather than breastmilk [8].

This suggests potential common biological mechanisms by which shortened gestation, delivery by caesarean section and a lack of breastmilk exposure may increase susceptibility to gut infections by disturbing or modifying gut microbiota and immune system homeostasis in early life. Previous population-based studies have investigated the independent associations of vaginal birth and breastmilk feeding with childhood gastroenteritis [9–11]. Few have examined the association with gestational age, especially those born around term, either preterm or early term (37–38 weeks gestation) and there is evidence these infants and children are at an increased risk of morbidity generally [12]. The combined risk of gastroenteritis associated with these birth characteristics is currently unknown, but such information is important given worldwide increasing rates of early planned birth and delivery by caesarean section [13–16], which are also associated with reduced rates of breastmilk feeding [17, 18]. Record linkage of large routinely collected population-based data with standardised clinical information provides a powerful approach to investigate the combined risk of gastroenteritis for multiple birth characteristics.

The aim of this study was to investigate the independent and combined associations of the mode and timing of birth and breastmilk feeding with gastroenteritis hospitalisations in early childhood.

## Methods

### Study population

The study population included all singleton live births of ≥33 weeks gestation from 2001 to 2011 in New South Wales (NSW), Australia. Stillbirths and births to non-NSW resident mothers were excluded as these births have no opportunity for follow-up through record linkage with hospital admissions in NSW. Infants with major congenital conditions, born before 33 weeks gestation, or twins and higher-order births were excluded as they have different risk profiles, outcomes and models of care [19]. Each child in the study population was followed from birth until the age of 6 years, death or the end of the study period (30 June 2012), whichever occurred first.

This study used linked birth, hospital and death records from the NSW Perinatal Data Collection (PDC), NSW Admitted Patient Data Collection (APDC) and Registry of Births, Deaths and Marriages Death Registrations (fact of death) respectively. The PDC is a population-based statutory collection covering all live births and stillbirths of at least 20 weeks gestation or, if gestational age is unknown, at least 400 grams birthweight. It contains information on maternal characteristics, pregnancy, labour and delivery factors, and infant outcomes. The APDC includes demographic and hospitalisation related data for every inpatient admitted to any public or private hospital in NSW. Diagnoses for each admission are coded according to the 10^th^ revision of the International Classification of Disease, Australian Modification (ICD-10-AM) [20]. Probabilistic record linkage of these data was performed by the NSW Centre for Health Record Linkage using methods described previously and only de-identified information was provided to the researchers [21]. The data sources used for this study require ethical and data custodian approval to access, link (by an independent and approved authority) and release for research. Approval for the record linkage and use of the data for research was obtained from the NSW Population and Health Services Research Ethics Committee and the appropriate data custodians.

### Mode of birth, timing of birth and infant feeding at discharge

The study factors of interest were mode of birth, gestational age (timing of birth) and infant feeding status at discharge from birth care. Mode of birth was defined using the combination of labour onset and type of birth (vaginal birth or caesarean section) and categorised as vaginal birth following spontaneous onset of labour, caesarean section following spontaneous onset of labour, vaginal birth following labour induction, caesarean section following labour induction, or pre-labour caesarean section. Gestational age is reported in completed weeks of gestation, as determined by the best clinical estimate including early ultrasound and last menstrual period. This was categorised as preterm (33–36 weeks), early-term (37–38 weeks) or term (39–42 weeks) birth. Infant feeding status at discharge from birth care is recorded using one or more of the following three categories: “breastfeeding”, “expressed breastmilk” or “infant formula”. These categories were used to create two independent groups: any breastmilk feeding (breastfeeding or expressed breastmilk feeding without infant formula) and formula-only feeding (infant formula without breastfeeding or expressed breastmilk feeding).

### Study outcome

The study outcome was hospital admissions for gastroenteritis, which we refer to as acute gastroenteritis (AGE). Primary or additional diagnoses for gastroenteritis (ICD-10-AM: A00-A09 or K52) were used to identify admissions. Inter-hospital transfers were treated as a continuation of an admission and not a new admission. AGE admissions within 7 days of a previous AGE admission were also treated as a single event.

### Statistical analysis

The proportion and number of children with none, one, or more than one AGE admission in the study period by maternal and perinatal characteristics were calculated. Cox proportional hazards regression was used to estimate the adjusted Hazard Ratios and 95 % confidence intervals for the independent and combined associations between the study exposures and first AGE hospitalisation with child age as the timescale and age at discharge from birth care as entry into observation. For censored individuals, age was recorded as the earliest of death, sixth birthday, or end of the study period (30 June 2012).

The covariates used in the study reflect known risk factors identified in the literature [4, 9, 12, 22]. Covariates included were: parity (primiparae or multiparae), maternal age (<20, 20–24, 25–29, 30–34, 35–39, 40+ years), country of birth (Australia-born or other), socio-economic status quintile (Australian Bureau of Statistics Socio-Economic Index For Areas – Index of Relative Socio-economic Advantage) [23], smoking during pregnancy, hypertensive disorders of pregnancy (gestational hypertension, preeclampsia or eclampsia), diabetes mellitus in pregnancy (gestational or pre-existing) [24, 25], baby’s sex, 5-minute Apgar score < 7, birthweight (standardised by gestational age and sex) [26], presence of AGE or other infection specific to the perinatal period (ICD-10-AM: P35-P39), admission to a Special Care Nursery (SCN) or Neonatal Intensive Care Unit (NICU) and infant birth admission length of stay (standardised by gestational age and type of birth using the study population). To account for the inclusion of rotavirus vaccination in the national immunisation program from 1 July 2007, year of birth was categorised as before July 2007 or July 2007 onwards. All covariates were adjusted for in the analysis except for admission to SCN or NICU and 5-minute Apgar score < 7 which were omitted from the model due to high co-linearity with birth at 33–36 weeks gestation. The assumption of proportional hazards was assessed using standard diagnostics and found to be reasonable.

Cox-regression was used to estimate the associations for the study exposures under variations of the study population and design, to assess the robustness of the main findings and for comparability with other studies of associations between birth factors and childhood hospitalisations. The variations investigated were: a sub-population of low risk liveborn singleton pregnancies (infants born at ≥37 weeks gestation, cephalic presenting, and a birthweight between the 10^th^ and 90^th^ percentiles for gestational age and sex, to women aged 20–34 years without medical conditions), using children with no hospital admissions only as the controls, using only a primary diagnosis of gastroenteritis, restricting to AGE hospitalisations within the first year of life or within the first two years of life (rather than six), and restricting to births from July 2007 onwards (universal rotavirus vaccination).

The level of missing information was minimal for all variables (<0.01 to 0.10 %), with the exception of infant feeding at discharge. Collection of infant feeding status began in mid-2006 and was only available for 51.8 % of births, within which 0.95 % were missing. As the per cent missing across all variables except infant feeding affected only 0.23 % of records, these were excluded. However, as infant feeding was an exposure of interest, it was imputed using a logistic model following recommendations in the literature (see Additional file 1). All analyses were performed using Stata 13.0 (StataCorp LP, TX, USA).

## Results

Of the 893,360 children included in the study, 28 % were delivered by caesarean section, 41 % were planned births (pre-labour caesarean or following labour induction), 27 % were born before 39 weeks gestation and 12 % were fed only formula in the birth admission (Table 1). There were 38,085 (4.3 %) children admitted to hospital for AGE once and 3,189 (0.4 %) more than once (7.7 % recurrence rate). The proportion of children admitted for AGE was higher for those delivered by caesarean section, born before 39 weeks gestation and fed only formula. Average follow-up per child was 4.37 years (standard deviation: 1.88), with a total follow-up time of 3,907,163 years. For the 41,274 children admitted, the median age at first AGE hospital admission was 1.43 years (Inter-quartile range 0.77 to 2.48 years).

**Table 1:** Maternal and perinatal characteristics for children admitted to hospital once and more than once for acute gastroenteritis, NSW 2001–2011

Variable	Number of AGE hospital admissions						Total (*N* = 893,360)	
	None (*N* = 852,086)		One (*N* = 38,085)		Two or more (*N* = 3189)			
	N	Row %^a^	N	Row %^a^	N	Row %^a^	N	Col. %^b^
Mode of birth								
Vaginal birth – spontaneous onset of labour	445,535	95.6	18,929	4.1	1469	0.3	465,933	52.2
Vaginal birth – labour induction	173,429	95.1	8220	4.5	722	0.4	182,371	20.4
Caesarean section – pre-labour	132,892	95.3	6017	4.3	561	0.4	139,470	15.6
Caesarean section – spontaneous onset of labour	57,537	94.9	2820	4.7	252	0.4	60,609	6.8
Caesarean section – labour induction	42,693	94.9	2099	4.7	185	0.4	44,977	5.0
Gestational age (weeks)								
33–36	36,508	94.4	1952	5.0	225	0.6	38,685	4.3
37–38	188,164	95.0	8985	4.5	834	0.4	197,983	22.2
39–42	627,414	95.5	27,148	4.1	2130	0.3	656,692	73.5
Maternal age (years)								
<20	31,020	92.8	2154	6.4	247	0.7	33,421	3.7
20–24	118,300	94.0	6902	5.5	676	0.5	125,878	14.1
25–29	235,736	95.1	11,235	4.5	926	0.4	247,897	27.8
30–34	282,186	95.8	11,464	3.9	853	0.3	294,503	33.0
35–39	153,271	96.4	5266	3.3	406	0.3	158,943	17.8
40+	31,573	96.5	1064	3.3	81	0.2	32,718	3.7
Primiparae	353,744	95.0	17,306	4.6	1468	0.4	372,518	41.7
Australian Born	598,374	95.0	29,024	4.6	2566	0.4	629,964	70.5
Socio-economic advantage								
1st Quintile (Highest)	173,516	96.1	6605	3.7	457	0.3	180,578	20.2
2nd Quintile	162,516	95.9	6420	3.8	501	0.3	169,437	19.0
3rd Quintile	178,752	95.5	7823	4.2	604	0.3	187,179	21.0
4th Quintile	175,417	94.9	8557	4.6	775	0.4	184,749	20.7
5th Quintile (Lowest)	161,885	94.4	8680	5.1	852	0.5	171,417	19.2
Smoking during pregnancy	126,685	94.2	7101	5.3	686	0.5	134,472	15.1
Diabetes	53,822	95.3	2420	4.3	220	0.4	56,462	6.3
Hypertension	74,126	94.5	3956	5.0	364	0.5	78,446	8.8
Baby’s sex - Male	436,304	95.2	20,085	4.4	1724	0.4	458,113	51.3
Year of birth								
Prior to 1 July 2007	473,607	93.6	29,552	5.8	2662	0.5	505,821	56.6
From 1 July 2007	378,479	97.7	8533	2.2	527	0.1	387,539	43.4
Birthweight z-score								
< -2	16,064	94.1	897	5.3	117	0.7	17,078	1.9
[-2,-1)	113,474	94.9	5570	4.7	482	0.4	119,526	13.4
[-1,0)	305,914	95.3	13,855	4.3	1154	0.4	320,923	35.9
[0,1)	281,683	95.5	12,173	4.1	994	0.3	294,850	33.0
[1,2)	108,087	95.7	4467	4.0	374	0.3	112,928	12.6
≥2	26,864	95.8	1123	4.0	68	0.2	28,055	3.1
Admission to SCN/NICU	111,483	94.2	6205	5.2	663	0.6	118,351	13.3
5 Minute Apgar Score < 7	10,947	94.5	584	5.0	59	0.5	11,590	1.3
Infection in birth admission^c^	17,475	94.1	991	5.3	109	0.6	18,575	2.1
Birth admission length of stay z-score								
< -1	81,018	96.3	2893	3.4	204	0.2	84,115	9.4
[-1,0)	346,181	95.7	14,527	4.0	1171	0.3	361,879	40.5
[0,1)	351,360	95.2	16,317	4.4	1371	0.4	369,048	41.3
≥ 1	73,527	93.9	4348	5.6	443	0.6	78,318	8.8
Formula-only feeding*								
Yes	52,360	96.4	1840	3.4	127	0.2	54,327	11.9
No	393,156	97.4	9936	2.5	660	0.2	403,752	88.1

*Complete cases only (*n* = 458,079), *SCN* Special Care Nursery, *NICU* Neonatal Intensive Care Unit, *Col.* Column

^a^Per cent of all children in the row. ^b^Per cent of all children in the study population. ^c^Includes AGE or ICD-10-AM: P35-P39

Compared to vaginal birth following spontaneous onset of labour, all other modes of birth were independently associated with a 12–23 % increased rate of AGE admission (Table 2). In general those modes of birth including delivery by caesarean section were similar with largely overlapping confidence intervals (19–23 % increased rate of admission), while vaginal birth following labour induction was intermediary to these modes of birth and vaginal birth following spontaneous onset of labour (12 % increased rate of admission). Adjusted associations for all variables are presented in the Additional file 2. The rate of AGE admission increased with decreasing gestational age. Birth at 37–38 weeks was associated with a 15 % increase in the rate of AGE admission (adjusted hazard ratio [aHR], 1.15; 95 % Confidence Interval [CI], 1.12–1.17), and for births at 33–36 weeks gestation was 23 % (aHR, 1.23; 95 % CI, 1.18–1.29). Infants fed only formula (aHR, 1.18; 95 % CI, 1.11–1.24) were also more likely to be admitted for AGE.

**Table 2:** Associations for age at first hospital admission for acute gastroenteritis by mode of birth, timing of birth and infant formula only at birth for the overall study and selected sub-populations, NSW 2001–2011^a^

	Study Population	Low-risk^c^	Healthy controls^d^	From 1 July 2007^e^	Primary^f^	1-year^g^	2-year^h^
Study population (N)	893,360	435,417	610,538	387,539	893,360	893,360	893,360
Children admitted (%)	4.6	4.6	6.8	2.3	4.1	1.6	3.0
	aHR (95 % CI)	aHR (95 % CI)	aHR (95 % CI)	aHR (95 % CI)	aHR (95 % CI)	aHR (95 % CI)	aHR (95 % CI)
Mode of birth							
Vaginal birth – spontaneous onset of labour	1.00 [Reference]	1.00 [Reference]	1.00 [Reference]	1.00 [Reference]	1.00 [Reference]	1.00 [Reference]	1.00 [Reference]
Vaginal birth – labour induction	1.12 (1.09–1.15)	1.12 (1.08–1.16)	1.15 (1.12–1.18)	1.10 (1.04–1.16)	1.13 (1.10–1.16)	1.18 (1.13–1.23)	1.14 (1.10–1.17)
Caesarean section – pre-labour	1.19 (1.16–1.23)	1.17 (1.11–1.22)	1.24 (1.20–1.27)	1.20 (1.13–1.27)	1.20 (1.16–1.23)	1.24 (1.18–1.31)	1.22 (1.17–1.26)
Caesarean section – spontaneous onset of labour	1.20 (1.16–1.25)	1.15 (1.08–1.22)	1.24 (1.20–1.29)	1.22 (1.13–1.33)	1.21 (1.16–1.26)	1.25 (1.17–1.34)	1.24 (1.18–1.30)
Caesarean section – labour induction	1.23 (1.18–1.29)	1.31 (1.22–1.41)	1.28 (1.22–1.34)	1.18 (1.07–1.29)	1.23 (1.17–1.29)	1.33 (1.23–1.43)	1.26 (1.20–1.33)
Gestational age (weeks)							
33–36	1.23 (1.18–1.29)	-	1.44 (1.37–1.50)	1.28 (1.16–1.40)	1.23 (1.17–1.29)	1.28 (1.19–1.38)	1.24 (1.17–1.31)
37–38	1.15 (1.12–1.17)	1.14 (1.10–1.19)	1.21 (1.18–1.24)	1.17 (1.11–1.23)	1.14 (1.11–1.17)	1.19 (1.15–1.24)	1.15 (1.12–1.19)
39–42	1.00 [Reference]	1.00 [Reference]	1.00 [Reference]	1.00 [Reference]	1.00 [Reference]	1.00 [Reference]	1.00 [Reference]
Formula-only feeding^b^	1.18 (1.11–1.24)	1.17 (1.08–1.26)	1.19 (1.14–1.25)	1.20 (1.14–1.28)	1.17 (1.11–1.24)	1.34 (1.25–1.44)	1.24 (1.17–1.31)

*CI* Confidence Interval, *aHR* Hazard Ratio. ^a^All models were adjusted for maternal country of birth, maternal smoking during pregnancy, socio-economic advantage, parity, diabetes, hypertension, baby’s sex, year of birth, birthweight, and length of stay and infections in the birth admission (AGE or ICD-10-AM: P35-P39), with the following exceptions; for the low-risk group diabetes and hypertension were not applicable and admission to a special care nursery or neonatal intensive care was able be included as preterm was not in the sub-group, for births occurring after 1 July 2007, the indicator for pre/post introduction of universal rotavirus vaccination was not applicable

^b^Reference category is absence of risk factor and the reported aHR includes uses imputed values

^c^Population restricted to low risk pregnancies: 10th–90th percentile birthweight for gestational age and sex, cephalic presenting, term births (≥37 weeks) to mothers aged 20–34 years without medical conditions

^d^Population restricted to children with one or more AGE hospital admissions or no hospital admissions

^e^Population restricted to children born after the inclusion of rotavirus vaccination in the Australian National Immunisation Program (1 July 2007)

^f^Age at first hospital admission with a primary diagnosis of AGE was used to define the event

^g^The age at first AGE hospital admission within the first year of life was used to define the event. For censored individuals, age was recorded as the earliest of death, first birthday, or end of the study period (30 June 2012)

^h^The age at first AGE hospital admission within the first two years of life was used to define the event. For censored individuals, age was recorded as the earliest of death, second birthday, or end of the study period (30 June 2012)

**Fig. 1:**
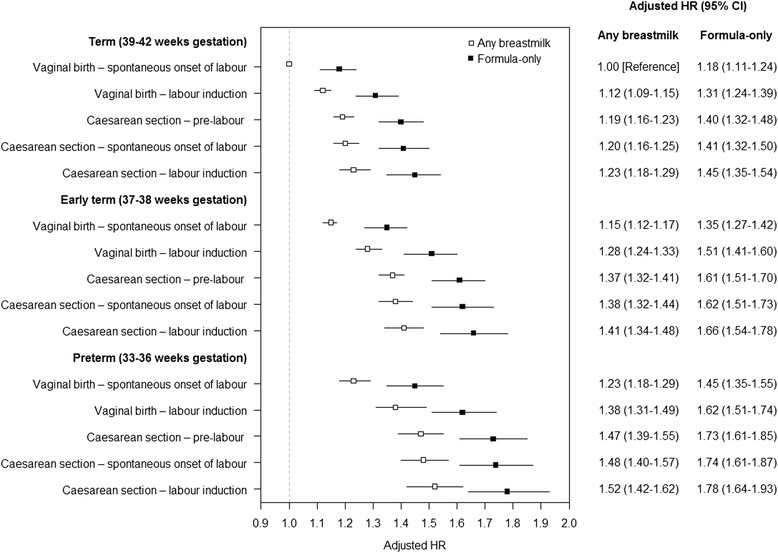
Combined associations for age at first hospital admission for acute gastroenteritis by mode of birth, timing of birth and infant formula only at birth, NSW 2001–2011. The reference category is vaginal, birth following spontaneous onset of labour at 39+ weeks gestation with breastmilk feeding at discharge. Associations adjusted for maternal country of birth, maternal smoking during pregnancy, socio-economic advantage, parity, diabetes, hypertension, baby’s sex, year of birth, birthweight, and length of stay and infections in the birth admission (AGE or ICD-10-AM: P35-P39)

Results for the combined associations are presented in Fig. 1. Compared with vaginal birth following spontaneous onset of labour at 39+ weeks gestation with any breastmilk feeding at discharge, early term births with formula-only feeding had an increased rate of AGE of 35 % and preterm birth a 45 % increased rate. Children born at early term with formula-only feeding had a higher rate of admission compared to preterm births that had some breastmilk feeding (35 % versus 23 %). For the other modes of birth with formula-only feeding, early term births had increased admission rates of 51–66 %, and preterm births had the highest rates ranging from 62–78 %. Within births 39+ weeks gestation, all modes of birth with formula-only feeding compared with vaginal birth following spontaneous onset of labour, had increased rates of admission by 31–45 %.

The results were mostly robust to changes in study population or design. Restriction to the first two years of life, low risk pregnancies, the period of rotavirus vaccination or a primary diagnosis of AGE provided generally similar adjusted hazard ratios to those for the selected study population (Table 2). The impact of formula-only feeding was stronger (34 % versus 18 %) when restricting to the first year of life. Restricting the control group to children with no hospital admissions provided a stronger association for gestational age (33–36 weeks, 44 % versus 23 %; 37–38 weeks, 21 % versus 15 %). For the adjusted final models and combined associations for the investigated changes in study population or design see Additional files 2 and 3.

## Discussion

This is the first population-based study to specifically investigate the combined effects of mode and timing of birth and breastmilk feeding at discharge from birth care on early childhood gastroenteritis hospital admissions. The results show an increased rate of admission in early childhood for being born before 39 weeks gestation, by modes of birth other than vaginal birth following spontaneous onset of labour and formula-only feeding at discharge from birth care. The combined effects highlight the benefit of normal birth and early breastmilk exposure, and are also suggestive of their impact on subsequent gastrointestinal health by possibly modifying or disturbing gut microbiota and immune system homeostasis. These findings are also pertinent in the context of increasing rates of caesarean section and early planned birth.

To our knowledge this is the first study to identify that children born preterm and early term, compared with children born at full term (39+ weeks gestation), had a 38–52 % and 28–41 % increased rate of AGE admission respectively, regardless of the mode of birth. Previous studies have demonstrated an increased rate of overall pediatric or respiratory hospitalisations for preterm and early term births [12, 27–29]. We also found for preterm and early term births, those with modes of birth other than vaginal birth following spontaneous onset of labour and formula-only feeding at discharge had even higher rates of AGE admission, 51–66 % and 62–78 % respectively. Interestingly, children born at early term had higher AGE admission rates than those born preterm who received any breastmilk feeding in the birth admission. Nevertheless, the most vulnerable group of children identified were those born preterm by modes of birth other than vaginal birth following the spontaneous onset of labour and who received formula-only at discharge from birth care.

The increased rate of AGE admission with decreasing gestational age may be explained in part by the under-preparedness of the newborns immune system, particularly in the gut epithelium, to respond to the initial microbial colonisation at birth. Differences in markers of immune function between infants born before and after 37 weeks gestation have been reported previously [30]. This may explain why vaginal births following labour induction had an increased rate of admission compared to those following the spontaneous onset of labour, as a decision has been made to deliver. Relative to the developmental trajectory of the infant, it may be that the necessary time required for the infant’s innate immune response to sufficiently mature has been circumvented.

Variation in gut microbial composition in infants by mode of birth (vaginal birth or caesarean section) and feeding status (breastmilk or formula) is well supported by clinical evidence [8, 31, 32]. These studies highlight that infants delivered by caesarean section or not exposed to breastmilk have less diverse gut microbiota dominated by “bad” bacteria [8, 31, 32]. The similarity of the adjusted associations for caesarean section regardless of the onset of labour is consistent with the theory of beneficial exposure to bacteria at the time of vaginal birth [9, 33]. The higher rate of admission for infants fed only formula is consistent with the idea that with minimal or no breastmilk exposure, there is a loss of the associated microbial and immunological benefits in early childhood. While our estimate (aHR, 1.18) was lower than other studies that have examined breastmilk feeding and AGE, these generally followed infants for the first 6–12 months of life, where a significant proportion of AGE admissions occur [34, 35]. The aHR of 1.34 from our analysis restricted to AGE admissions in the first year of life is similar to these studies, suggesting a potentially stronger association earlier in life.

Previous population-based record-linkage studies of term births and without breastmilk feeding information found, as we did, an increased risk of AGE for children delivered by caesarean section [9, 10]. However, with the known association between caesarean section and difficulty initiating breastfeeding [18], the combined association for both factors is of particular interest. We found that despite term birth, children delivered by caesarean section and formula-only feeding at discharge from birth care had a 40–45 % increased rate of admission. Even for infants with breastmilk exposure, caesarean section was still associated with increased rates of AGE admission (19–23 %). This suggests that for infants delivered by caesarean section, breastfeeding initiation and duration are important factors for reducing the risk of AGE in early childhood and alternative methods of exposure to beneficial bacteria at the time of birth are required.

Changes in clinical obstetric practice have seen an increase in rates of planned birth before 39 weeks gestation and caesarean section worldwide [13–16], and the adverse impact of caesarean section on breastfeeding is well-established [18]. As these trends relate to factors hypothesised to have a common biological basis for affecting the risk of acute gastroenteritis, the impact of a continuation of these patterns on AGE should not be underestimated. Although, the increasing recognition of the potential harms of early elective births and subsequent introduction of clinical guidelines, policies and interventions to reduce labour induction or pre-labour caesarean section for non-medical reasons before 39–40 weeks gestation may counter these trends [36–40].

As planned birth before 39 weeks increases the perceptions of women about what constitutes normal birth is also likely to be altered. Recent studies have demonstrated that almost one in four women believed that a baby was full-term at 34–36 weeks gestation, that one in two believed full term was 37–38 weeks and more than 90 % believed it was safe to deliver before 39 weeks [41, 42]. Another study demonstrated that many women had little knowledge of the benefits or risks of a caesarean section, yet almost half indicated that a caesarean section without medical indication should be given on request [43]. Given the multi-faceted changes in practice and attitudes towards earlier births and interventions, similar studies to ours are vital to assess the impact on long term outcomes to inform clinicians, and women and their families.

The strengths of this study are that it is a large population-based cohort, using data and variables with demonstrated accuracy and validity [44, 45]. Using a large population-based cohort also reduces the impact of genetic diversity and enables complete ascertainment of hospital admissions. The mode and timing of birth and infant feeding at discharge were all statistically significant and consistently so for all analyses (coefficient *p*-values were typically < 0.001), so it is unlikely our findings are due to chance. However, some caution is warranted as some of the associations for the study factors are small and by chance a statistically significant association may be found when performing many comparisons. Using available administrative data has some limitations, as not all potentially relevant characteristics such as diet, environment or antibiotic use during pregnancy and early childhood could be investigated. Despite the lack of information on long term breastfeeding outcomes such as the duration of exclusive breastfeeding, previous studies have found in-hospital formula supplementation is associated with early cessation of exclusive or any breastmilk feeding post-discharge [46–48]. Identified cases of acute gastroenteritis were based on hospital admissions only which represent the severe end of the clinical spectrum and do not include mild cases that may be treated through out-patient facilities or primary care services.

## Conclusions

We have shown using a large population-based record-linkage study that the rate of acute gastroenteritis hospitalisations in early childhood are increased for births by caesarean section or induction of labour, before 39 weeks gestation and for infants fed only formula at discharge from birth care. The combined effects of these factors highlight the benefit of normal birth and early breastmilk exposure, for reducing the risk of gastroenteritis hospitalisation in early childhood. These previously unknown combined effects represent useful information against a backdrop of increasing rates of caesarean section and early planned birth, and their potential impact on gut microbiota and immune system homeostasis.

### Availability of data and materials

The data used in this study cannot be shared by the Authors due to the use and release of the data being subject to data custodian and ethics approval and conditions that require the data only be used for approved research, by approved persons directly involved in the project and following the completion of a confidentiality undertaking prior to the information being released.

## Additional files

Additional file 1: “Imputation.pdf” summarises the imputation approach for formula-only feeding at discharge from birth care, and compares the associations for the study factors and all covariates between the imputation and complete case analysis. (DOC 92 kb) Additional file 2: “All adjusted associations.pdf” summarises the adjusted associations for the study factors and all covariates used in the adjustment for the main and additional study populations. (DOC 84 kb) Additional file 3: “Additional combined adjusted associations.pdf” summarises the combined associations for the study factors for all additionally investigated study populations. (DOC 443 kb)

## Abbreviations

AGE: acute gastroenteritis
aHR: adjusted Hazard Ratio
APDC: admitted patient data collection
CI: confidence interval
ICD-10-AM: International Classification of Disease, 10th revision, Australian Modification
NICU: Neonatal Intensive Care Unit
NSW: New South Wales
PDC: perinatal data collection
SCN: special care nursery
