# Supplementary material for: Gestational age, mode of birth and breastmilk feeding all influence acute early childhood gastroenteritis: a record-linkage cohort study
Source: BMC Pediatr. 2016 Apr 27;16:55. doi: 10.1186/s12887-016-0591-0 (PMC4847338; doi:10.1186/s12887-016-0591-0)
Supplement: Additional file 1: — “Imputation.pdf” summarises the imputation approach for formula-only feeding at discharge from birth care, and compares the associations for the study factors and all covariates between the imputation and complete case analysis. (DOC 92 kb) [file 12887_2016_591_MOESM1_ESM.doc]

**Additional file 1 - Imputation**

The logistic imputation model used all study variables, outcome variables represented by the binary indicator for event or censoring and the Nelson-Aalen estimator of the cumulative hazard, and additional auxiliary variables.^1^ Auxiliary variables are those associated with breastfeeding status at discharge, but which were not part of this study. These included; weeks gestational at first antenatal visit, presentation at birth, season of birth, placenta praevia or placental abruption, post-partum hemorrhage, public or private care, diagnosis codes for lactation problems/supervision or drug and alcohol and chronic maternal medical conditions other than diabetes or hypertension.^2, 3^ We initially used 50 imputations following recommendations in the literature and then performed a further 50 imputations to ensure that the Monte Carlo error of all estimated coefficients, including the combined linear associations, was less than 10% of the standard error.^4^ The observed and imputed distributions for breastfeeding were compared for all variables and indicated reasonable agreement (not shown). Cox-regression results for a complete case analysis (mid-2006 to 2011) and the model using the imputed information for breastfeeding were compared (Supplementary Figure 1) and showed similar associations for all variables, particularly for the study factors of interest: mode of birth, gestational age and formula-only feeding. Based on these findings, results using the imputed values of formula-only feeding are reported.

**References**

1. White IR, Royston P. Imputing missing covariate values for the Cox model. Statistics in Medicine 2009;28:1982-1998.

2. Sterne JA, White IR, Carlin JB, et al. Multiple imputation for missing data in epidemiological and clinical research: potential and pitfalls. BMJ 2009;338:b2393.

3. Lee KJ, Carlin JB. Multiple imputation for missing data: fully conditional specification versus multivariate normal imputation. Am J Epidemiol 2010;171:624-32.

4. White IR, Royston P, Wood AM. Multiple imputation using chained equations: Issues and guidance for practice. Statistics in Medicine 2011;30:377-399.

**Supplementary Figure 1:** Comparison of adjusted hazards ratios for complete case and imputation, NSW 2001-2011


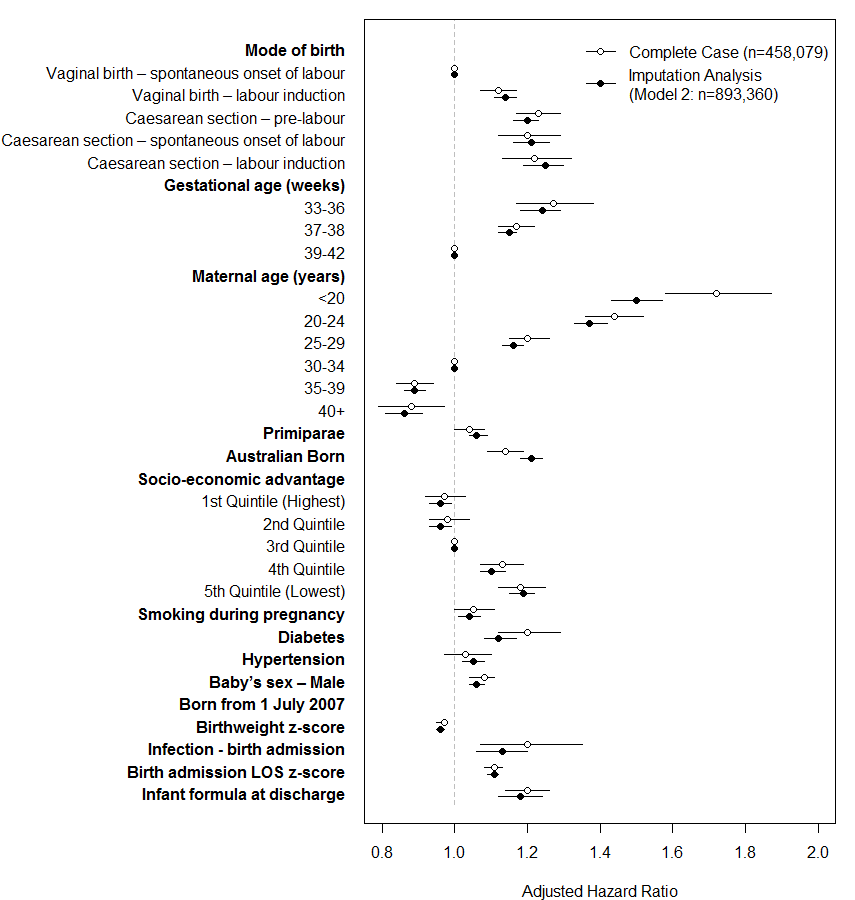


Infection in the birth admission includes either gastroenteritis or or other infection specific to the perinatal period (ICD-10-AM: P35-P39). LOS = Length of Stay.
