# Supplementary material for: Gestational age, mode of birth and breastmilk feeding all influence acute early childhood gastroenteritis: a record-linkage cohort study
Source: BMC Pediatr. 2016 Apr 27;16:55. doi: 10.1186/s12887-016-0591-0 (PMC4847338; doi:10.1186/s12887-016-0591-0)
Supplement: Additional file 2: — “All adjusted associations.pdf” summarises the adjusted associations for the study factors and all covariates used in the adjustment for the main and additional study populations. (DOC 84 kb) [file 12887_2016_591_MOESM2_ESM.doc]

**Additional file 2 - Adjusted associations for age at first hospital admission for acute gastroenteritis for the overall study and selected sub-populations, NSW 2001-2011***

|  | **Study Population** | **Low-risk^1^** | **Healthy controls^2^** | **From 1 July 2007^3^** | **Primary^4^** | **1-year^5^** | **2-year^6^** |
| --- | --- | --- | --- | --- | --- | --- | --- |
| Study population (N) | 893,360 | 435,417 | 610,538 | 387,539 | 893,360 | 893,360 | 893,360 |
| Children admitted (%) | 4.6 | 4.6 | 6.8 | 2.3 | 4.1 | 1.6 | 3.0 |
|  | **aHR (95% CI)** | **aHR (95% CI)** | **aHR (95% CI)** | **aHR (95% CI)** | **aHR (95% CI)** | **aHR (95% CI)** | **aHR (95% CI)** |
| **Mode of birth** |  |  |  |  |  |  |  |
| Vaginal birth – spontaneous onset of labour | 1 [Reference] | 1 [Reference] | 1 [Reference] | 1 [Reference] | 1 [Reference] | 1 [Reference] | 1 [Reference] |
| Vaginal birth – labour induction | 1.12 (1.09-1.15) | 1.12 (1.08-1.16) | 1.15 (1.12-1.18) | 1.10 (1.04-1.16) | 1.13 (1.10-1.16) | 1.18 (1.13-1.23) | 1.14 (1.10-1.17) |
| Caesarean section – pre-labour | 1.19 (1.16-1.23) | 1.17 (1.11-1.22) | 1.24 (1.20-1.27) | 1.20 (1.13-1.27) | 1.20 (1.16-1.23) | 1.24 (1.18-1.31) | 1.22 (1.17-1.26) |
| Caesarean section – spontaneous onset of labour | 1.20 (1.16-1.25) | 1.15 (1.08-1.22) | 1.24 (1.20-1.29) | 1.22 (1.13-1.33) | 1.21 (1.16-1.26) | 1.25 (1.17-1.34) | 1.24 (1.18-1.30) |
| Caesarean section – labour induction | 1.23 (1.18-1.29) | 1.31 (1.22-1.41) | 1.28 (1.22-1.34) | 1.18 (1.07-1.29) | 1.23 (1.17-1.29) | 1.33 (1.23-1.43) | 1.26 (1.20-1.33) |
| **Gestational age (weeks)** |  |  |  |  |  |  |  |
| 33-36 | 1.23 (1.18-1.29) | [Not applicable] | 1.44 (1.37-1.50) | 1.28 (1.16-1.40) | 1.23 (1.17-1.29) | 1.28 (1.19-1.38) | 1.24 (1.17-1.31) |
| 37-38 | 1.15 (1.12-1.17) | 1.14 (1.10-1.18) | 1.21 (1.18-1.24) | 1.17 (1.11-1.23) | 1.14 (1.11-1.17) | 1.19 (1.15-1.24) | 1.15 (1.12-1.19) |
| 39-42 | 1 [Reference] | 1 [Reference] | 1 [Reference] | 1 [Reference] | 1 [Reference] | 1 [Reference] | 1 [Reference] |
| **Maternal age (years)** |  |  |  |  |  |  |  |
| <20 | 1.50 (1.43-1.57) | [Not applicable] | 1.61 (1.54-1.69) | 1.83 (1.66-2.03) | 1.48 (1.41-1.56) | 2.07 (1.92-2.24) | 1.70 (1.61-1.80) |
| 20-24 | 1.37 (1.33-1.42) | 1.41 (1.35-1.46) | 1.44 (1.39-1.48) | 1.45 (1.36-1.55) | 1.38 (1.33-1.42) | 1.64 (1.56-1.73) | 1.47 (1.41-1.52) |
| 25-29 | 1.16 (1.13-1.19) | 1.16 (1.12-1.19) | 1.19 (1.16-1.22) | 1.24 (1.17-1.31) | 1.16 (1.13-1.19) | 1.29 (1.23-1.35) | 1.20 (1.17-1.24) |
| 30-34 | 1 [Reference] | 1 [Reference] | 1 [Reference] | 1 [Reference] | 1 [Reference] | 1 [Reference] | 1 [Reference] |
| 35-39 | 0.89 (0.86-0.92) | [Not applicable] | 0.87 (0.85-0.90) | 0.90 (0.84-0.96) | 0.88 (0.85-0.91) | 0.84 (0.80-0.89) | 0.86 (0.82-0.89) |
| 40+ | 0.86 (0.81-0.91) | [Not applicable] | 0.84 (0.79-0.89) | 0.89 (0.79-1.00) | 0.87 (0.81-0.92) | 0.79 (0.71-0.88) | 0.82 (0.76-0.88) |
| **Primiparae**^ | 1.06 (1.04-1.09) | 1.05 (1.02-1.08) | 1.05 (1.02-1.07) | 1.03 (0.98-1.08) | 1.10 (1.07-1.12) | 0.90 (0.87-0.94) | 1.02 (0.99-1.05) |
| **Australian Born**^ | 1.21 (1.18-1.24) | 1.24 (1.20-1.28) | 1.32 (1.28-1.35) | 1.10 (1.05-1.15) | 1.24 (1.21-1.27) | 1.31 (1.25-1.36) | 1.25 (1.21-1.29) |
| **Socio-economic advantage** |  |  |  |  |  |  |  |
| 1st Quintile (Highest) | 0.96 (0.93-0.99) | 1.00 (0.96-1.05) | 0.97 (0.94-1.00) | 1.00 (0.93-1.07) | 0.94 (0.91-0.98) | 1.03 (0.97-1.09) | 1.04 (0.99-1.08) |
| 2nd Quintile | 0.96 (0.93-0.99) | 0.95 (0.91-1.00) | 0.95 (0.92-0.98) | 1.02 (0.95-1.09) | 0.94 (0.91-0.97) | 0.99 (0.94-1.05) | 1.00 (0.96-1.04) |
| 3rd Quintile | 1 [Reference] | 1 [Reference] | 1 [Reference] | 1 [Reference] | 1 [Reference] | 1 [Reference] | 1 [Reference] |
| 4th Quintile | 1.10 (1.07-1.14) | 1.09 (1.05-1.14) | 1.10 (1.07-1.14) | 1.15 (1.08-1.22) | 1.09 (1.06-1.12) | 1.14 (1.08-1.20) | 1.11 (1.07-1.16) |
| 5th Quintile (Lowest) | 1.19 (1.15-1.22) | 1.17 (1.12-1.22) | 1.21 (1.18-1.25) | 1.19 (1.11-1.27) | 1.19 (1.15-1.22) | 1.25 (1.19-1.32) | 1.21 (1.17-1.26) |
| **Smoking during pregnancy**^ | 1.04 (1.01-1.07) | 1.08 (1.04-1.12) | 1.08 (1.05-1.11) | 1.05 (0.99-1.12) | 1.03 (1.00-1.06) | 1.16 (1.11-1.21) | 1.08 (1.05-1.12) |
| **Diabetes**^ | 1.12 (1.08-1.17) | [Not applicable] | 1.12 (1.07-1.16) | 1.22 (1.13-1.32) | 1.11 (1.07-1.16) | 1.10 (1.03-1.18) | 1.13 (1.07-1.18) |
| **Hypertension**^ | 1.05 (1.01-1.08) | [Not applicable] | 1.06 (1.03-1.10) | 1.03 (0.95-1.10) | 1.05 (1.02-1.09) | 1.06 (1.00-1.12) | 1.06 (1.02-1.10) |
| **Baby’s sex – Male**^ | 1.06 (1.04-1.08) | 1.06 (1.03-1.09) | 1.23 (1.21-1.26) | 1.07 (1.02-1.11) | 1.04 (1.02-1.06) | 1.10 (1.07-1.14) | 1.06 (1.04-1.09) |
| **Born from 1 July 2007**^ | 0.55 (0.54-0.56) | 0.54 (0.52-0.56) | 0.48 (0.47-0.50) | [Not applicable] | 0.51 (0.49-0.52) | 0.69 (0.67-0.72) | 0.58 (0.56-0.59) |
| **Birthweight z-score**^#^ | 0.96 (0.95-0.97) | 0.96 (0.94-0.98) | 0.96 (0.95-0.97) | 0.96 (0.94-0.98) | 0.96 (0.95-0.97) | 0.98 (0.96-0.99) | 0.96 (0.95-0.97) |
| **SCN/NICU admission**^ | [Omitted] | 1.11 (1.05-1.16) | [Omitted] | [Omitted] | [Omitted] | [Omitted] | [Omitted] |
| **5 Minute Apgar Score < 7**^ | [Omitted] | [Omitted] | [Omitted] | [Omitted] | [Omitted] | [Omitted] | [Omitted] |
| **Infection - birth admission**^ŧ | 1.13 (1.06-1.20) | 1.21 (1.10-1.32) | 1.17 (1.10-1.24) | 1.19 (1.04-1.37) | 1.12 (1.05-1.20) | 1.20 (1.09-1.33) | 1.16 (1.08-1.25) |
| **Birth admission length of stay z-score**^#^ | 1.11 (1.10-1.12) | 1.11 (1.09-1.13) | 1.15 (1.13-1.16) | 1.11 (1.08-1.14) | 1.10 (1.09-1.12) | 1.09 (1.07-1.12) | 1.11 (1.09-1.12) |
| **Formula-only feeding**^ | 1.18 (1.11-1.24) | 1.17 (1.08-1.26) | 1.19 (1.14-1.25) | 1.20 (1.14-1.28) | 1.17 (1.11-1.24) | 1.34 (1.25-1.44) | 1.24 (1.17-1.31) |

CI = Confidence Interval, HR = Hazard Ratio, SCN = Special Care Nursery, NICU = Neonatal Intensive Care Unit.

* Adjusted for all variables with reported Hazard Ratios in the column.

^ Reference category is absence of risk factor.

ŧ Infections in the birth admission is either AGE or other infection specific to the perinatal period (ICD-10-AM: P35-P39)

# The HR is for a one unit change in the z-score which represents the change in the rate of AGE admission for a difference of one standard deviation.

1. Population restricted to low risk pregnancies: 10th-90th percentile birthweight for gestational age and sex, cephalic presenting, term births (≥37 weeks) to mothers aged 20-34 years without medical conditions.

2. Population restricted to children with one or more AGE hospital admissions or no hospital admissions.

3. Population restricted to children born after the inclusion of rotavirus vaccination in the Australian National Immunisation Program (1 July 2007).

4. Age at first hospital admission with a primary diagnosis of AGE was used to define the event.

5. The age at first AGE hospital admission within the first year of life was used to define the event. For censored individuals, age was recorded as the earliest of death, first birthday, or end of the study period (30 June 2012).

6. The age at first AGE hospital admission within the first two years of life was used to define the event. For censored individuals, age was recorded as the earliest of death, second birthday, or end of the study period (30 June 2012).
