# Supplementary material for: Gestational age, mode of birth and breastmilk feeding all influence acute early childhood gastroenteritis: a record-linkage cohort study
Source: BMC Pediatr. 2016 Apr 27;16:55. doi: 10.1186/s12887-016-0591-0 (PMC4847338; doi:10.1186/s12887-016-0591-0)
Supplement: Additional file 3: — “Additional combined adjusted associations.pdf” summarises the combined associations for the study factors for all additionally investigated study populations. (DOC 443 kb) [file 12887_2016_591_MOESM3_ESM.doc]

**Additional file 3 - Combined adjusted associations for age at first hospital admission for each study variation, NSW 2001-2011**

**Figure 3a.** Combined adjusted associations for age at first hospital admission for acute gastroenteritis by mode of birth, timing of birth and infant formula only at discharge from birth care for low-risk pregnancies, NSW 2001-2011*


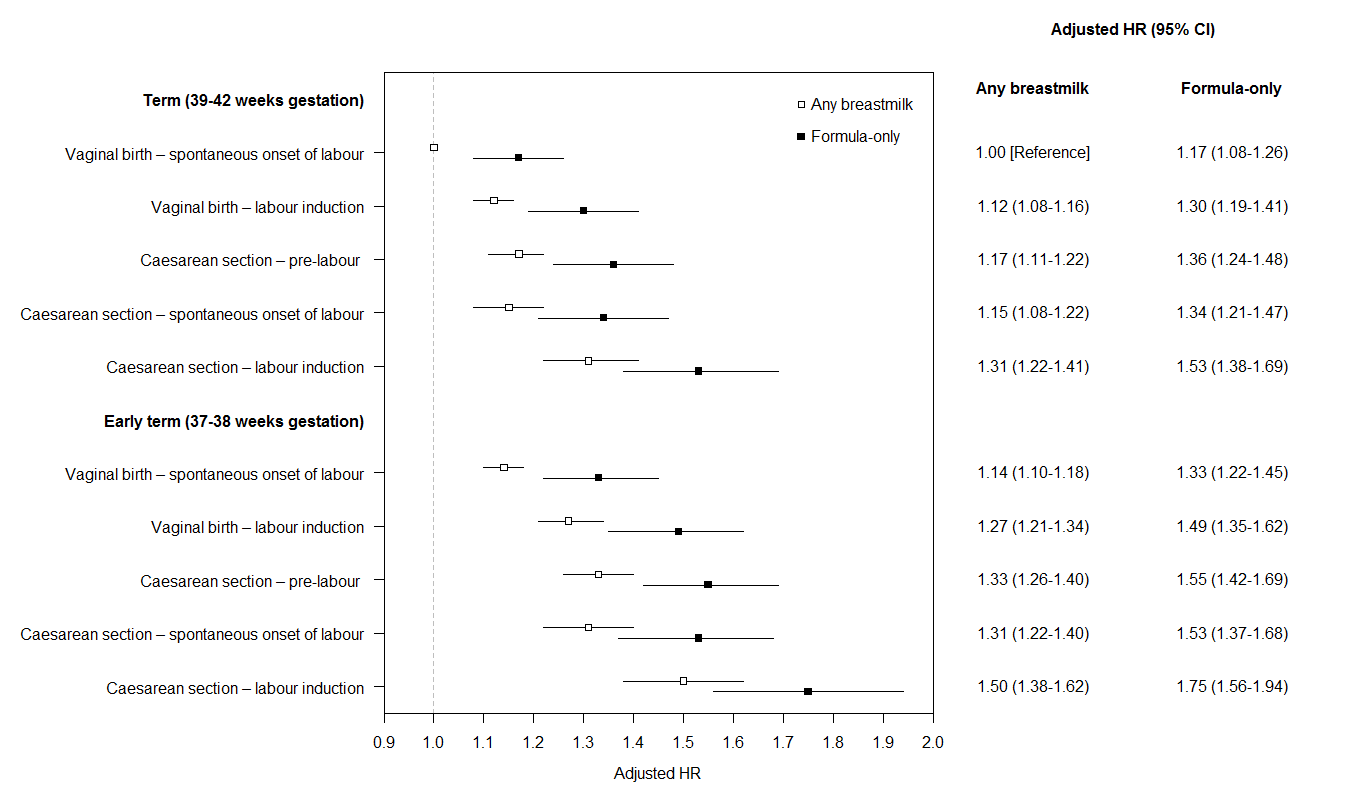


*Model adjusted for maternal country of birth, maternal smoking during pregnancy, socio-economic advantage, parity, baby’s sex, year of birth, birthweight, admission to a Special Care Nursery or Neonatal Intensive Care Unit and length of stay and infections in the birth admission (AGE or ICD-10-AM: P35-P39). Low risk pregnancies: 10th-90th percentile birthweight for gestational age and sex, cephalic presenting, term births (≥37 weeks) to mothers aged 20-34 years without medical conditions.

**Figure 3b.** Combined adjusted associations for age at first hospital admission for acute gastroenteritis by mode of birth, timing of birth and infant formula only at discharge from birth care using children never admitted to hospital as controls, NSW 2001-2011*


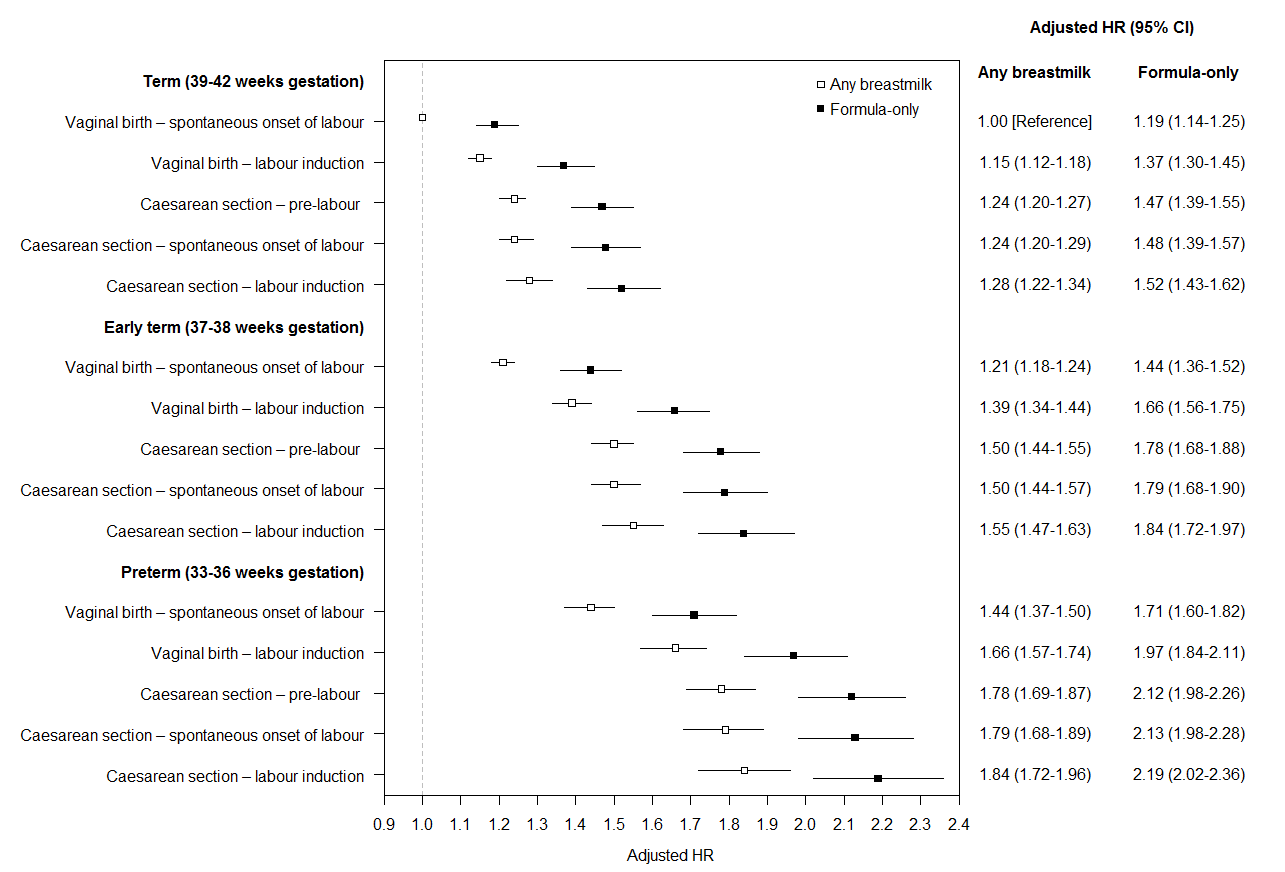


*Model adjusted for maternal country of birth, maternal smoking during pregnancy, socio-economic advantage, parity, diabetes, hypertension, baby’s sex, year of birth, birthweight, and length of stay and infections in the birth admission (AGE or ICD-10-AM: P35-P39).

**Figure 3c.** Combined adjusted associations for age at first hospital admission for acute gastroenteritis by mode of birth, timing of birth and infant formula only at discharge from birth care during the period of universal rotavirus vaccination, NSW July 2007-2011*


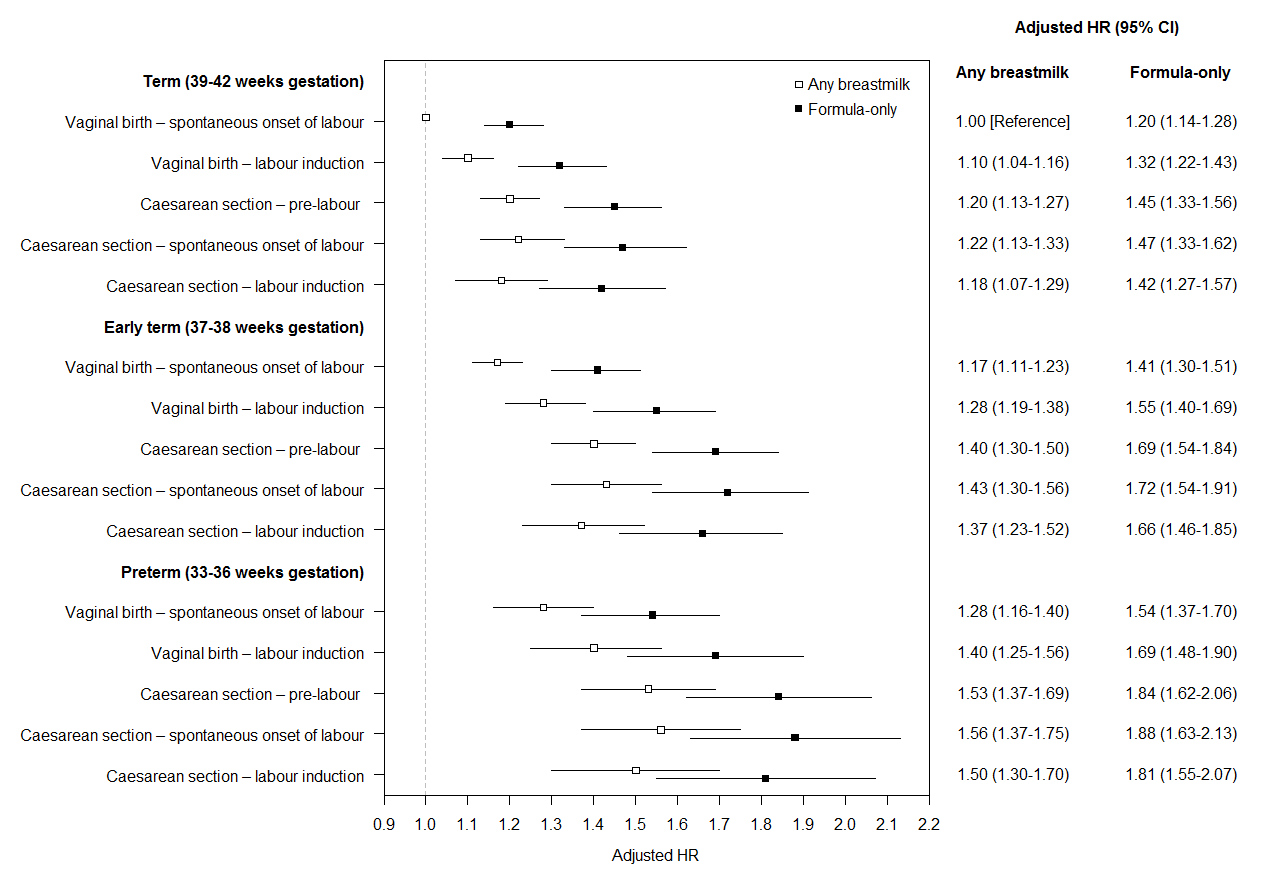


*Model adjusted for maternal country of birth, maternal smoking during pregnancy, socio-economic advantage, parity, diabetes, hypertension, baby’s sex, birthweight, and length of stay and infections in the birth admission (AGE or ICD-10-AM: P35-P39).

**Figure 3d.** Combined adjusted associations for age at first hospital admission for acute gastroenteritis by mode of birth, timing of birth and infant formula only at discharge from birth care using primary diagnosis only to define AGE, NSW 2001-2011*


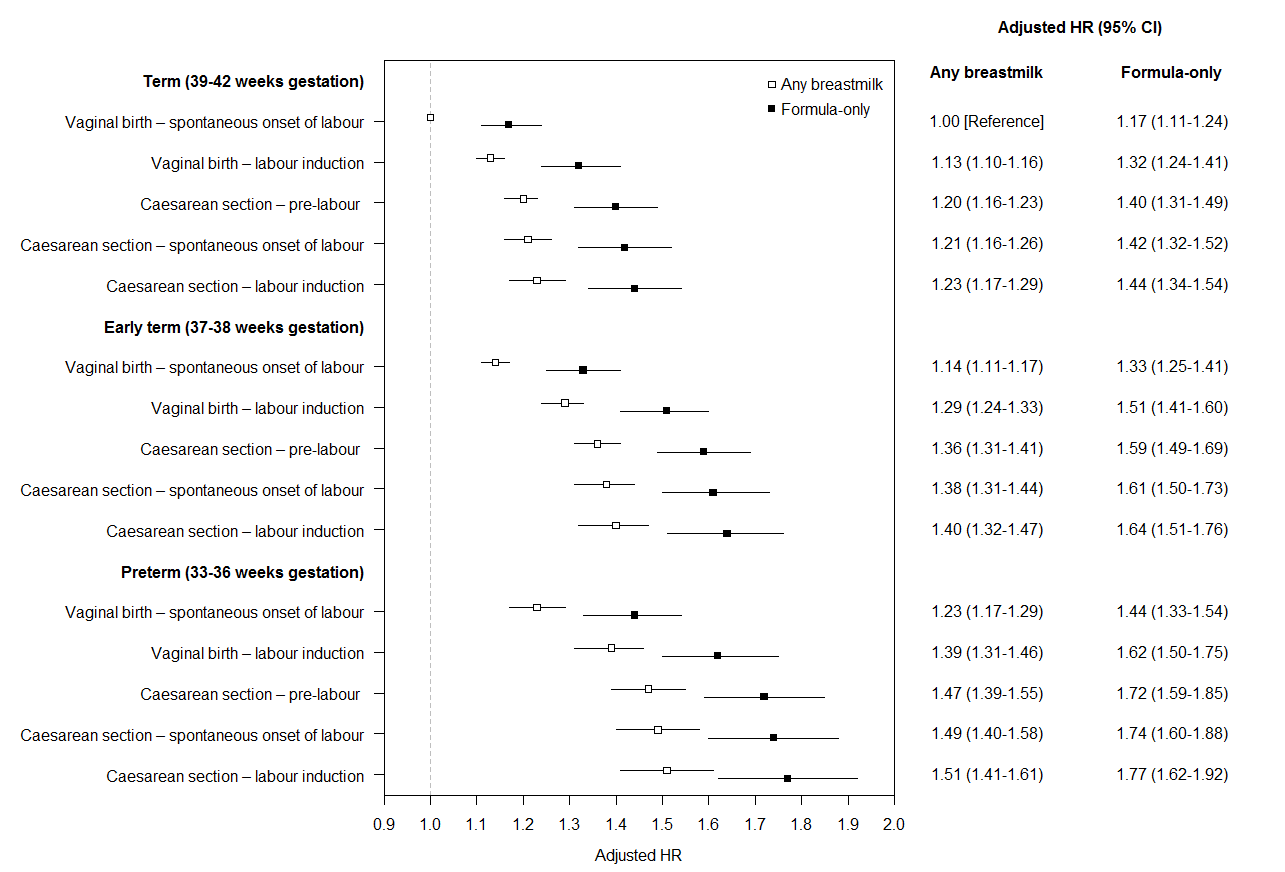


*Model adjusted for maternal country of birth, maternal smoking during pregnancy, socio-economic advantage, parity, diabetes, hypertension, baby’s sex, year of birth, birthweight, and length of stay and infections in the birth admission (AGE or ICD-10-AM: P35-P39).

**Figure 3e.** Combined adjusted associations for age at first hospital admission within the first year of life for acute gastroenteritis by mode of birth, timing of birth and infant formula only at discharge from birth care, NSW 2001-2011*


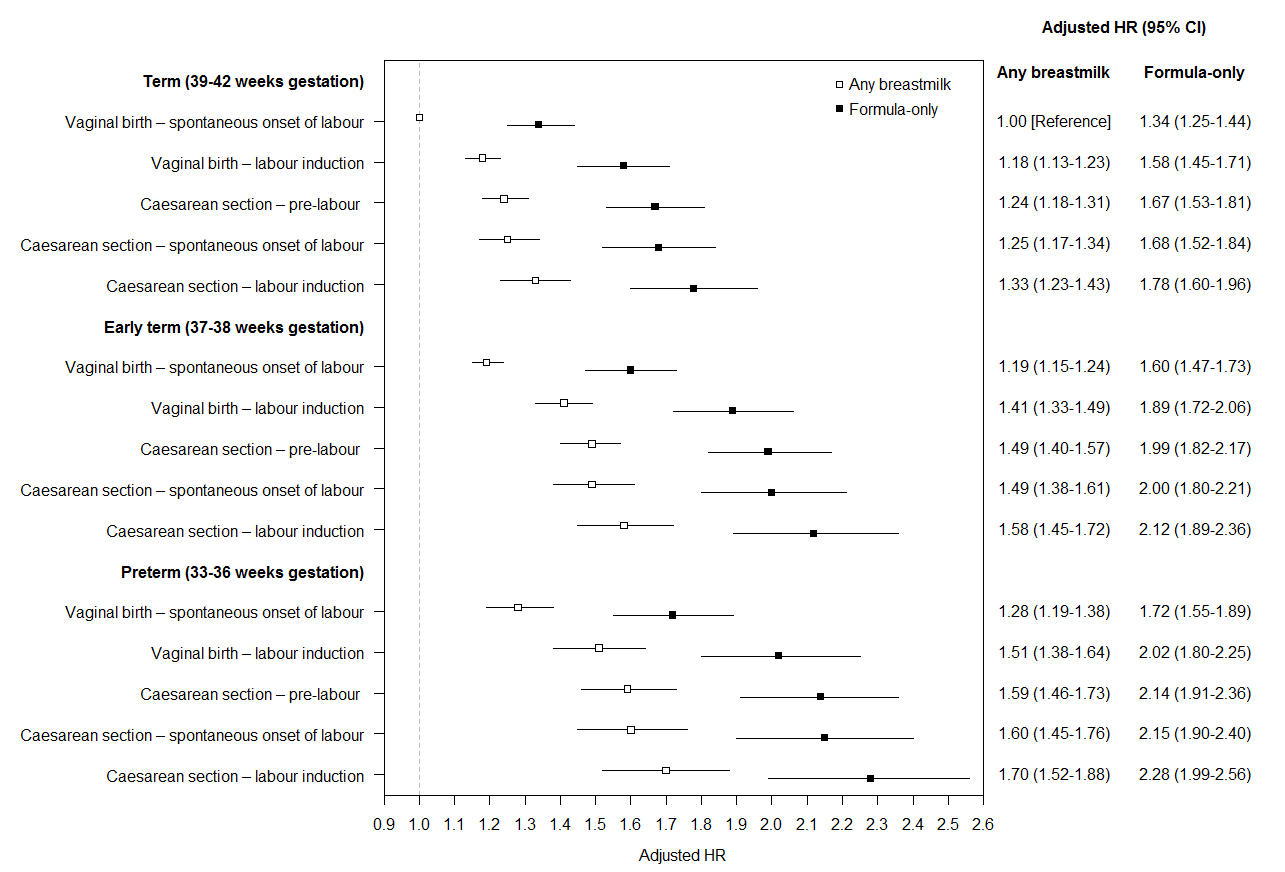


*Model adjusted for maternal country of birth, maternal smoking during pregnancy, socio-economic advantage, parity, diabetes, hypertension, baby’s sex, year of birth, birthweight, and length of stay and infections in the birth admission (AGE or ICD-10-AM: P35-P39).

**Figure 3f.** Combined adjusted associations for age at first hospital admission within the first two years of life for acute gastroenteritis by mode of birth, timing of birth and infant formula only at discharge from birth care, NSW 2001-2011*


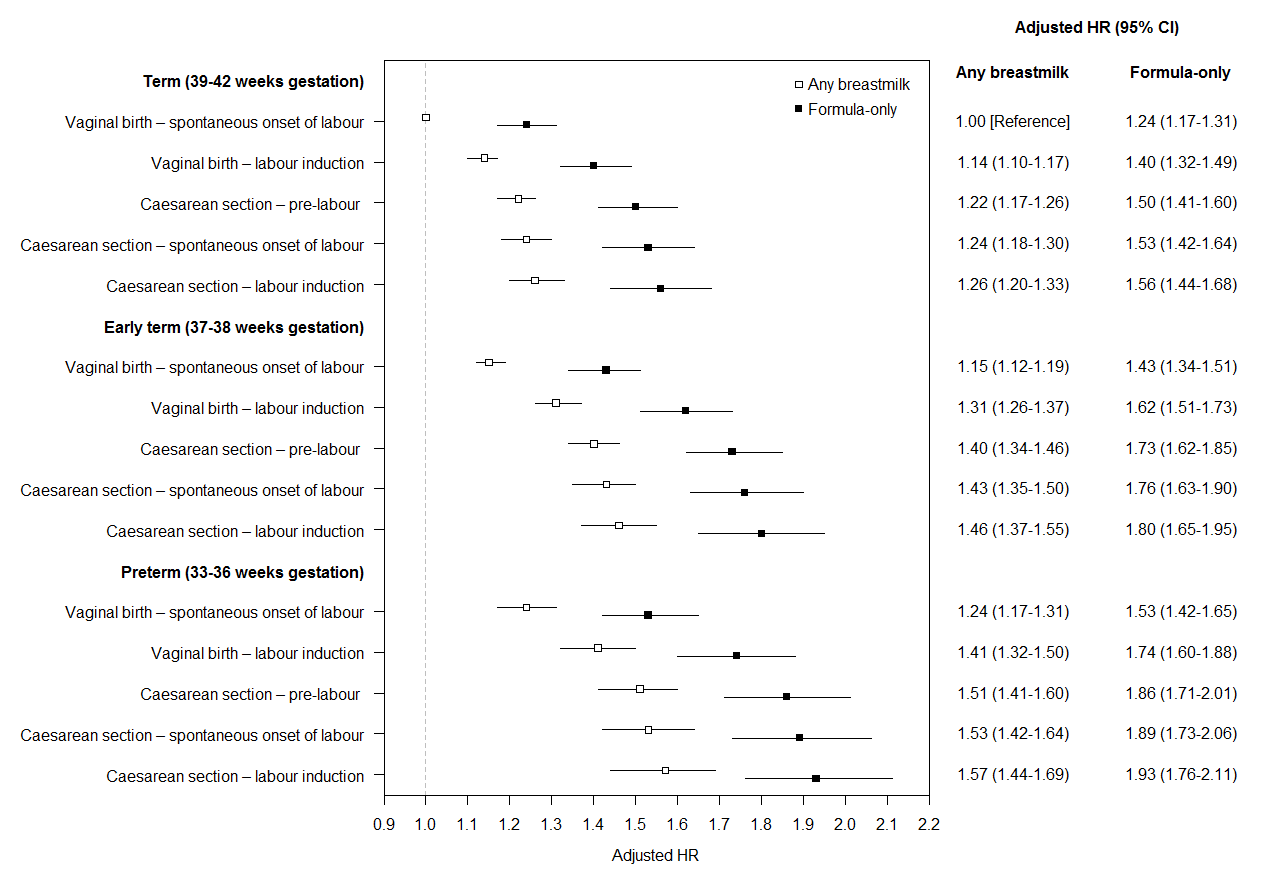


*Model adjusted for maternal country of birth, maternal smoking during pregnancy, socio-economic advantage, parity, diabetes, hypertension, baby’s sex, year of birth, birthweight, and length of stay and infections in the birth admission (AGE or ICD-10-AM: P35-P39).
